# Supplementary material for: Structural basis for antibody cross-neutralization of Dengue and Zika viruses
Source: Commun Biol. 2026 Mar 10;9:568. doi: 10.1038/s42003-026-09805-6 (PMC13106624; doi:10.1038/s42003-026-09805-6)
Supplement: Supplementary file 2 — Supplementary Information [file 42003_2026_9805_MOESM2_ESM.pdf]

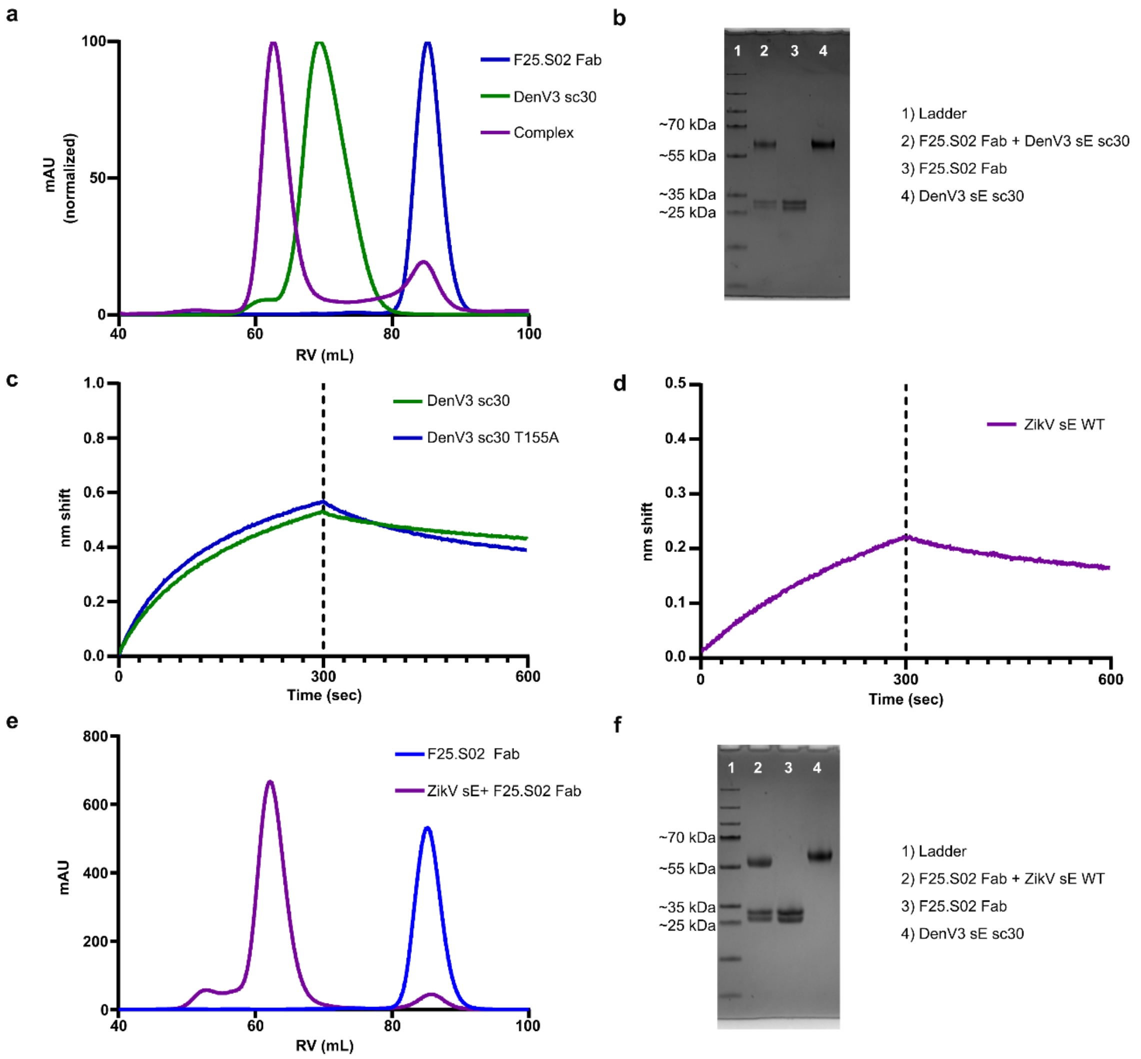

Supplementary Figure 1. **Purification and binding assays of F25.S02 Fab to DenV3 and ZikV sE dimers**. a) SEC traces of F25.S02 Fab, DenV3 sE sc30, and the complex of the two. mAu was normalized as F25 Fab expressed at a much higher level. b) SDS-PAGE of SEC purified peak of F25.S02 Fab + DenV3 sE sc30 complex and the individual components. Gel was run under reducing conditions. c) BLI data of F25.S02 Fab binding to DenV3 sE sc30 and N153 glycan deletion. Binding data showed that presence of lack of glycan did not affect binding. d) BLI data of F25.S02 Fab binding to ZikV sE WT dimer. e) SEC traces of F25.S02 Fab and coexpression of ZikV sE WT + F25.S02 Fab. f) SDS-PAGE of SEC purified peak of F25.S02 Fab + ZikV sE complex and the individual components. DenV3 sE sc30 was included to show relative size of sE monomer. Gel was run under reducing conditions.

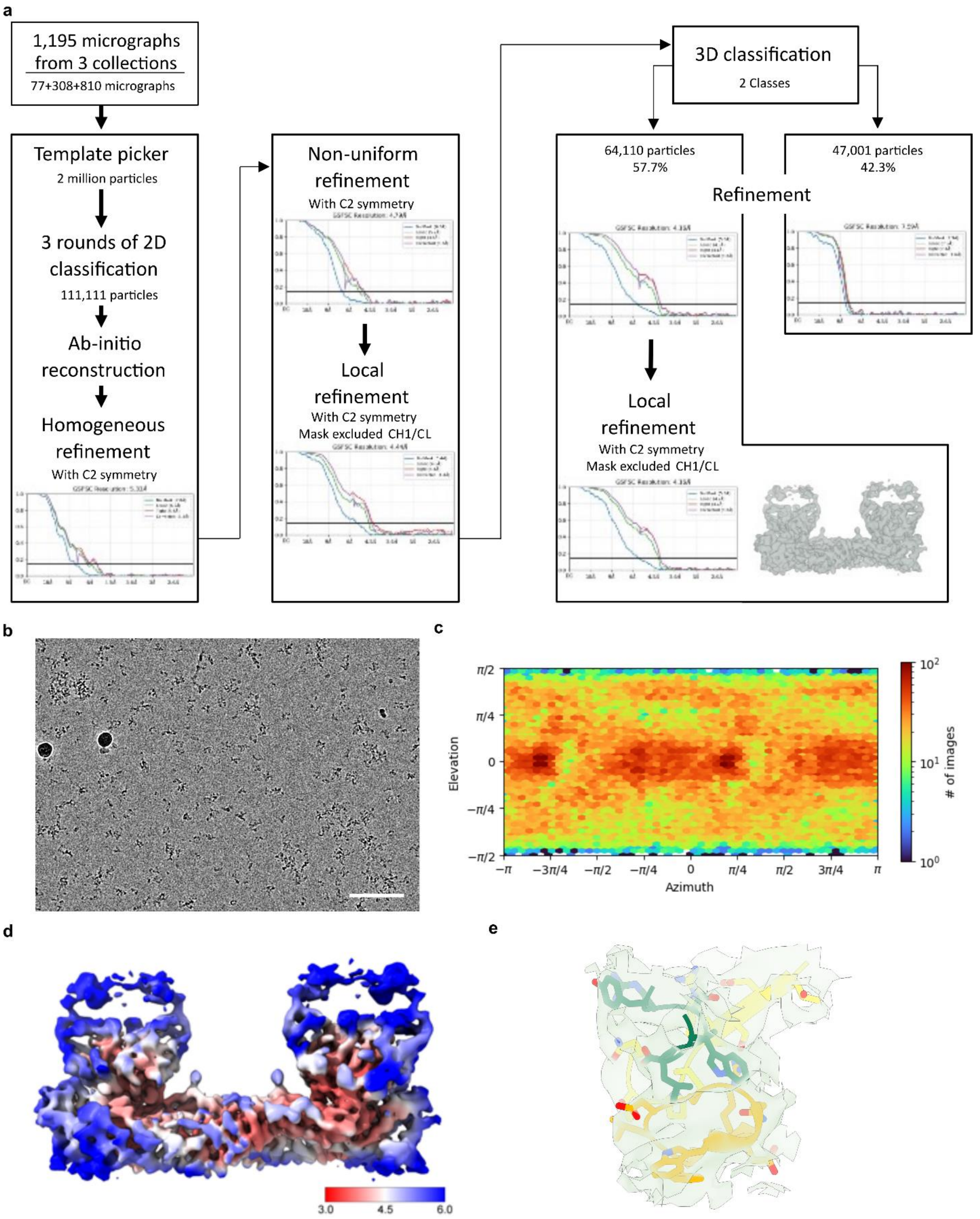

Supplementary Figure 2. **CryoEM data collection and processing**. a) Data processing workflow for DenV3 sE sc30 bound to F25.S02 structure. b) Representative motion corrected micrograph from data collection. Scale bar is 100 nm. c) View direction distribution plot of particles in 3D reconstruction. d) 3D reconstruction map colored by local resolution. Scale bar represents resolution in Å. e) Model fit image to 3D reconstruction. Focused on fusion loop and CDRH3 of F25.S02. Map is cut off within 3 Å of model.

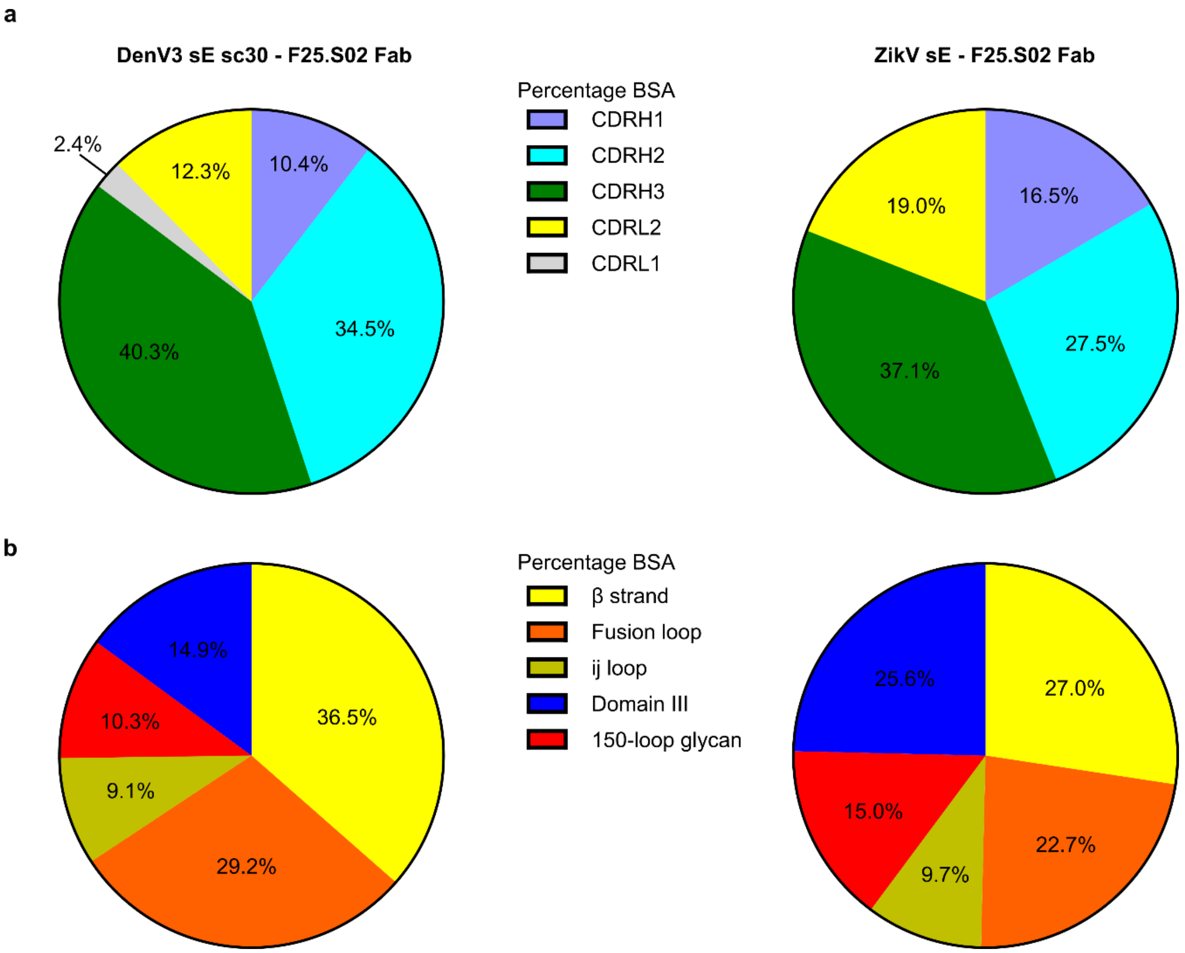

Supplementary Figure 3. Pie chart of buried surface area (BSA) of binding interface. a) BSA component of each complementary determining region of the heavy and light chain of F25.S02. b) BSA component of each region of the sE protein. BSA was calculated using the PDBePISA server.

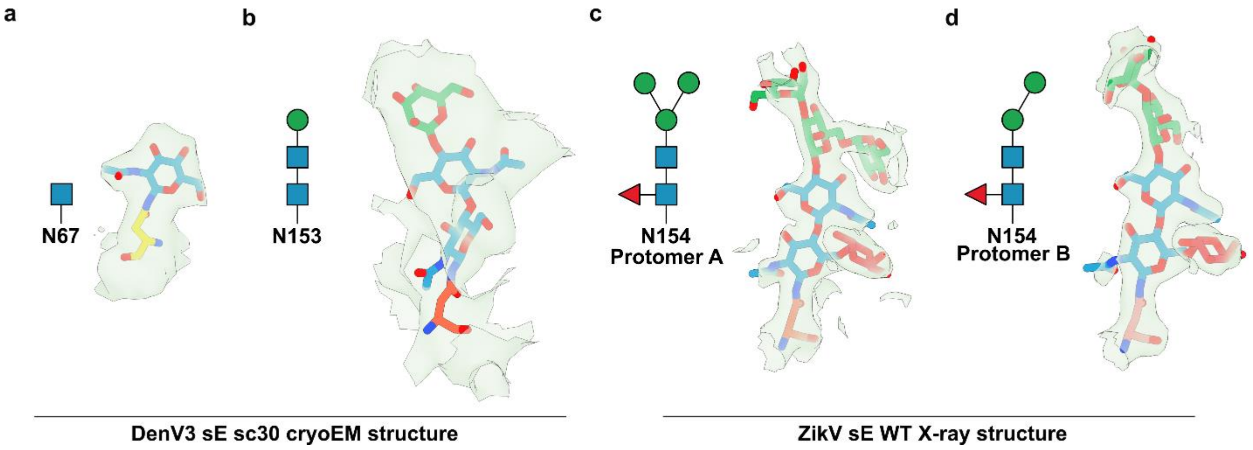

Supplementary Figure 4. CryoEM map and electron density map around glycans. a-b) Reconstructed cryoEM map within 3 Å of glycan at position 67 (A) and 153 (B) of DenV3. C2 symmetry was used in reconstruction so the map for glycans on each protomer is identical. c-d) Electron density contoured to 1.0 RMSD within 3 Å of the N154 glycan of ZikV. The glycan diagram for each is shown with blue squares, red triangles, and green circles representing N-acetylglucosamine, fucose, and mannose, respectively.

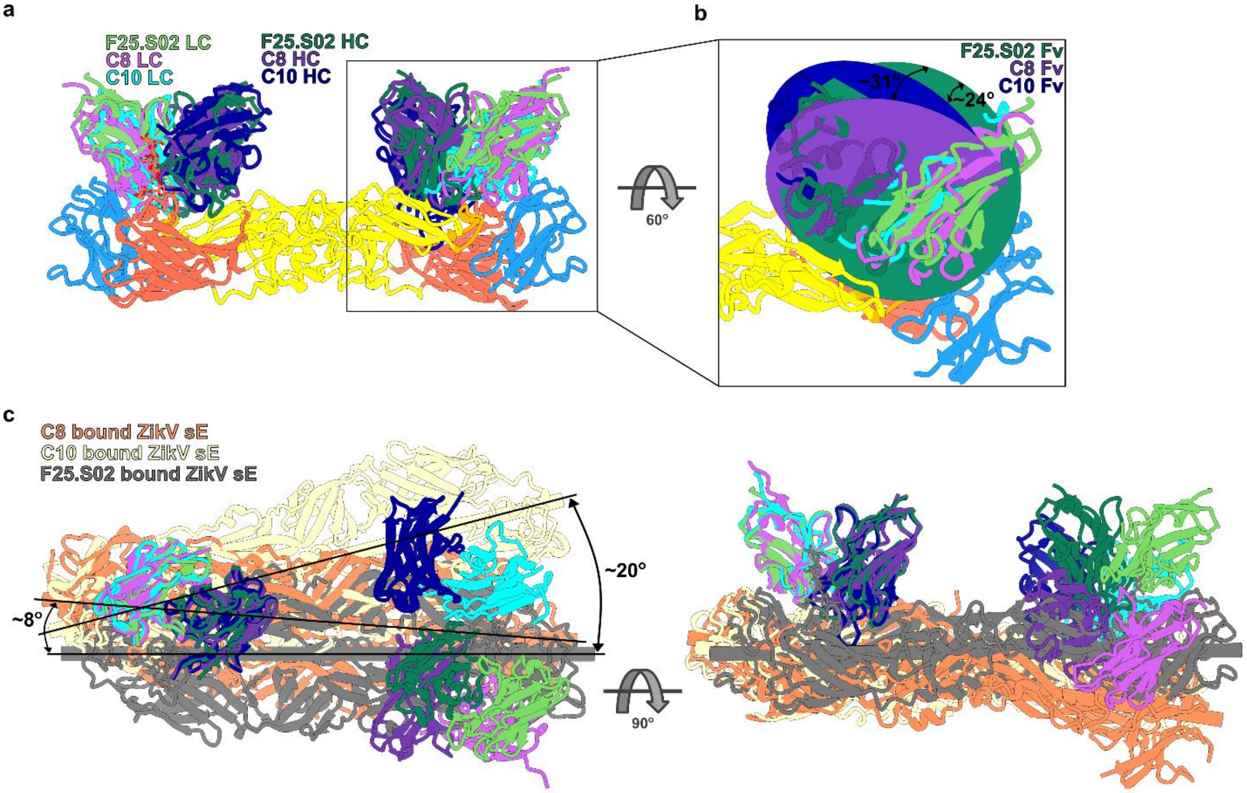

Supplemental Figure 5. Differences in binding angles between F25.S02, C8, and C10. a) Using the same alignment from Fig4a, **b)** a plane representing the center of mass of each of the bnAbs Fv was generated. The sE for C8 and C10 was omitted for simplicity. **c)** Alignment of the Fv domains of each of F25.S02, C8, and C10 shows the different angles that each Fv uses to engage with the sE protein (left). The sE protein is flexible with a hinge between domains I and II (right).

	BSA of Fab (Å²)			BSA of CDRs (Å²)						BSA with Glycan	
DenV3 sE sc30 - F25.S02 Fab	Heavy Chain (%)	Light Chain (%)	Total	H1	H2	H3	L1	L2	L3	Heavy chain	Light Chain
	1010 (87%)	147 (13%)	1157	95	314	367	22	112	0	201	13
ZikV sE - F25.S02 Fab											
Protomer A - Fab (chain H-L)	1130 (81%)	269 (19%)	1399	173	291	392	0	200	0	219	69
Protomer B - Fab (chain C-D)	1078 (81%)	245 (19%)	1323	174	287	387	0	199	0	198	46
Average	1104 (81%)	257 (19%)	1361	174	289	390	0	200	0	209	58

	BSA sE dimer (Å²)					
	Reference Protomer			Opposite Protomer		total
DenV3 sE sc30 - F25.S02 Fab	β strand	Fusion loop	ij loop	150-loop glycan	Domain III	
	337	270	84	95	138	924
ZikV sE - F25.S02 Fab						
Protomer A - Fab (chain H-L)	292	251	117	167	269	1096
Protomer B - Fab (chain C-D)	299	245	94	161	263	1062
Average	296	248	106	164	266	1079

Supplementary Table 1. Buried surface area (BSA) for DenV3 sE sc30 - F25.S02 Fab and ZikV sE - F25.S02 Fab binding. BSA values of F25.S01 Fab and the sE proteins of DenV3 and ZikV in Å^2 calculated using the PDBePISA server [1]. The BSA values are broken down by heavy and light chain, each complementary determining region (CDR), and interaction with the 150-loop glycan. Only one binding site is reported for the DenV3 sE sc30 - F25.S02 Fab as binding sites are equivalent as 2-fold symmetry was used to generate the final model. Two sites for ZikV sE - F25.S02 Fab are reported as crystallization contacts caused minor differences in the total BSA of each site.

Domains of sE	DenV3 sE sc30 - F25.S02 Fab					ZikV sE - F25.S02 Fab (H-L)					ZikV sE - F25.S02 Fab (C-D)				
	sE	Protomer	dist (Å)	F25.S02	CDR	sE	Protomer	dist (Å)	F25.S02	CDR	sE	Protomer	dist (Å)	F25.S02	CDR
β strand	T 70 [OG1]	A	2.67	F 54 [O]	H2	S 70 [N]	A	3.59	G 55 [O]	H2	S 70 [N]	B	3.19	G 55 [O]	H2
	T 70 [OG1]	A	2.89	G 55 [C]	H2	S 70 [O]	A	3.63	T 56 [OG1]	H2	S 70 [O]	B	3.75	T 56 [OG1]	H2
	T 70 [OG1]	A	2.89	T 56 [N]	H2	S 70 [CB]	A	3.35	F 54 [O]	H2	S 70 [CB]	B	2.97	F 54 [O]	H2
	D 71 [CA]	A	3.50	T56 [OG1]	H2	D 71 [CA]	A	3.51	T 56 [OG1]	H2	D 71 [CA]	B	3.65	T 56 [OG1]	H2
	S 72 [O]	A	3.52	T56 [OG1]	H2	S 72 [N]	A	3.47	T 56 [OG1]	H2	S 72 [N]	B	3.60	T 56 [OG1]	H2
	S 72 [O]	A	3.06	S 100B [OG]	H3	S 72 [O]	A	2.75	S 100B [OG]	H3	S 72 [O]	B	2.76	S 100B [OG]	H3
						S 72 [OG]	A	2.62	T 56 [OG1]	H2	S 72 [OG]	B	2.76	T 56 [OG1]	H2
	R 73 [CB]	A	3.31	R 100A [O]	H3	R 73 [CA]	A	3.28	R 100A [O]	H3	R 73 [CA]	B	3.33	R 100A [O]	H3
						R 73 [NH1]	A	3.60	S 100B [OG]	H3	R 73 [NH1]	B	3.78	S 100B [OG]	H3
						R 73 [NH2]	A	3.60	N 99 [ND2]	H3	R 73 [NH2]	B	3.78	N 99 [ND2]	H3
	74 C [N]	A	3.38	R 100A [O]	H3	C 74 [N]	A	2.80	R 100A [O]	H3	C 74 [N]	B	2.69	R 100A [O]	H3
						C 74 [SG]	A	3.71	S 100B [C]	H3	C 74 [SG]	B	3.70	S 100B [C]	H3
						C 74 [SG]	A	3.69	A 100C [N]	H3	C 74 [SG]	B	3.70	A 100C [N]	H3
	74 C [SG]	A	3.10	A 100D [OD1]	H3	C 74 [SG]	A	3.57	D 100D [OD1]	H3	C 74 [SG]	B	3.57	D 100D [OD1]	H3
	77 Q [O]	A	3.05	R 100A [NH1]	H3	Q 77 [OE1]	A	3.16	R 100A [NH1]	H3	Q 77 [OE1]	B	3.10	R 100A [NH1]	H3
	77 Q [NE2]	A	3.21	Y 100 [CE2]	H3						Q 77 [OE1]	B	3.82	Y 100 [CE2]	H3
fusion loop															
	V 97 [CG2]	A	3.80	F 54 [CE1]	H2	V 97 [CG1]	A	3.48	F 54 [CE1]	H2	V 97 [CG1]	B	3.49	F 54 [CE1]	H2
	R 99 [NH2]	A	3.13	F 54 [CE2]	H2	R 99 [NE]	A	3.33	F 54 [CE2]	H2	R 99 [NE]	B	3.61	F 54 [CE2]	H2
	R 99 [NH2]	A	3.25	M 52 [CE]	H2	R 99 [NH2]	A	3.72	M 52 [CE]	H2	R 99 [NH2]	B	3.71	M 52 [CE]	H2
						R 99 [NH2]	A	3.20	S 100B [O]	H3	R 99 [NH2]	B	3.04	S 100B [O]	H3
						W 101 [CA]	A	3.81	L 100E [CD1]	H3	W 101 [CA]	B	3.75	L 100E [CD1]	H3
	G 102 [O]	A	2.83	S 31 [OG]	H1	G 102 [N]	A	3.53	W 100F [CH2]	H3	G 102 [N]	B	3.87	W 100F [CH2]	H3
	D 103 [C]	A	3.14	W 100F [CH2]	H3	N 103 [C]	A	3.40	W 100F [CH2]	H3	N 103 [C]	B	3.34	W 100F [CH2]	H3
	D 103 [O]	A	3.01	M 52 [CE]	H2	N 103 [CB]	A	3.73	S 31 [CB]	H1	N 103 [CB]	B	3.60	S 31 [CB]	H1
	G 104 [N]	A	3.14	W 100F [CH2]	H3	G 104 [CA]	A	3.33	W 100F [CE2]	H3	G 104 [CA]	B	3.36	W 100F [CE2]	H3
						G 104 [O]	A	3.14	A 100C [C]	H3	G 104 [O]	B	3.14	A 100C [N]	H3
	G 104 [O]	A	3.74	D 100D [N]	H3	G 104 [O]	A	3.05	D 100D [N]	H3	G 104 [O]	B	3.14	D 100D [N]	H3
	G 104 [O]	A	3.12	L 100E [N]	H3	G 104 [O]	A	3.02	L 100E [N]	H3	G 104 [O]	B	2.99	L 100E [N]	H3
						C 105 [CA]	A	3.47	D 100D [OD1]	H3	C 105 [CA]	B	3.53	D 100D [OD1]	H3
	C 105 [O]	A	3.51	L 100E [CD1]	H3	C 105 [O]	A	3.53	L 100E [CD1]	H3	C 105 [O]	B	3.42	L 100E [CD1]	H3
	D 106 [N]	A	3.35	D 100D [OD2]	H3	G 106 [N]	A	3.03	D 100D [OD1]	H3	G 106 [N]	B	3.10	D 100D [OD1]	H3
	D 106 [CA]	A	3.32	L 100E [CD1]	H3	G 106 [N]	A	3.58	L 100E [CG]	H3	G 106 [N]	B	3.50	L 100E [CG]	H3
	D 106 [CB]	A	3.73	D 100D [OD2]	H3										
150-loop	H 149 [CB]	B	3.78	Y 32 [OH]	H1	G 150 [O]	B	3.18	Y 32 [OH]	H1	G 150 [CA]	A	3.77	Y 32 [OH]	H1
	E 154 [OE1]	B	3.55	S 31 [CB]	H1	V 153 [O]	B	2.88	S 30 [OG]	H1	G 150 [C]	A	3.82	Y 32 [OH]	H1
	T 155 [CG2]	B	3.25	T 28 [CB]	FW1	V 153 [C]	B	3.73	S 30 [OG]	H1	G 150 [O]	A	3.03	Y 32 [OH]	H1
	T 155 [CG2]	B	3.55	S 30 [CB]	H1	V 153 [O]	B	3.86	S 30 [CB]	H1	V 153 [O]	A	3.74	S 30 [CB]	H1
						V 153 [CG1]	B	3.31	S 30 [OG]	H1	V 153 [O]	A	3.68	S 30 [OG]	H1
						V 153 [CG1]	B	3.57	S 31 [OG]	H1	V 153 [CG1]	A	3.69	S 31 [OG]	H1
						T 156 [CG2]	B	3.70	G 27 [O]	FW1	T 156 [CG2]	A	3.62	G 27 [CA]	FW1
						T 156 [CG2]	B	3.71	T 28 [CG2]	FW1					
ij loop						K 251 [O]	A	3.48	F 54 [CE1]	H2	K 251 [O]	B	3.29	F 54 [CE1]	H2
	K 245 [CD]	A	3.75	I 53 [O]	H2	R 252 [CG]	A	3.23	I 53 [O]	H2	R 252 [NE]	B	3.29	I 53 [O]	H2
						R 252 [NH2]	A	3.56	P 52A [O]	H2					
						R 252 [NH2]	A	2.75	E 73 [OE2]	FW3	R 252 [NH1]	B	3.61	E 73 [OE2]	FW3
Domain III						K 316 [CE]	B	3.86	L 100E [CD1]	H3	K 316 [CE]	A	3.77	L 100E [CD1]	H3
	K 307 [CE]	B	3.71	Y 49 [OH]	L2	T 315 [O]	B	2.89	K 53 [NZ]	L2	T 315 [O]	A	2.95	K 53 [NZ]	L2
	K 307 [O]	B	3.85	K 53 [CE]	L2	E 370 [OE1]	B	3.13	K 54 [NH2]	L2	E 370 [OE1]	A	3.19	K 54 [NH2]	L2
	K 307 [NZ]	B	3.38	R 54 [O]	L2	E 370 [OE2]	B	2.75	K 54 [NH1]	L2	E 370 [OE2]	A	3.06	K 54 [NH1]	L2
	K 308 [NZ]	B	3.67	K 53 [NZ]	L2	E 370 [OE2]	B	3.56	D 60 [CB]	L2					
	K 308 [CE]	B	3.73	L 100E [CD2]	H3	N 371 [O]	B	3.50	S 56 [OG]	L2					
	E 361 [OE1]	B	3.71	S 56 [CB]	L2	N 371 [ND2]	B	3.11	R 54 [O]	L2	N 371 [ND2]	A	2.91	R 54 [O]	L2
						K 373 [NZ]	B	3.09	Y 49 [OH]	L2	K 373 [NZ]	A	2.62	R 54 [O]	L2
						K 373 [NZ]	B	3.25	R 54 [O]	L2					
											K 373 [NZ]	A	3.75	P 55 [CA]	L2
153/154 glycan	NAG 154-1 [O3]	B	3.84	PCA 1 [N]	HC-FW1	NAG 154-1 [O3]	B	3.19	PCA 1 [OXT]	HC-FW1	NAG 154-1 [O3]	A	3.19	PCA 1 [OXT]	HC-FW1
	NAG 154-1 [C8]	B	3.32	S 30 [OG]	H1						NAG 154-1 [C8]	A	3.69	S 30 [OG]	H1
	NAG 154-1 [O7]	B	2.85	Y 32 [OH]	H1	NAG 154-1 [C8]	B	3.46	Y 32 [CE2]	H1	NAG 154-1 [C8]	A	3.46	Y 32 [CE2]	H1
						NAG 154-1 [O3]	B	3.11	R 94 [NH2]	HC-FW3	NAG 154-1 [O3]	A	3.49	R 94 [NH1]	HC-FW3
	NAG 154-2 [O5]	B	3.74	PCA 1 [OXT]	HC-FW1	NAG 154-2 [O5]	B	3.67	PCA 1 [OXT]	HC-FW1	NAG 154-2 [O5]	A	3.64	PCA 1 [OXT]	HC-FW1
	NAG 154-2 [O6]	B	3.41	R 94 [NH2]	HC-FW3	NAG 154-2 [O5]	B	3.90	R 94 [NH2]	HC-FW3	NAG 154-2 [O6]	A	3.54	R 94 [NH2]	HC-FW3
											NAG 154-2 [O7]	A	3.60	L 102 [CD2]	HC-FW4
	NAG 154-2 [O3]	B	3.87	V 2 [CG2]	HC-FW1	NAG 154-2 [O7]	B	3.52	V 2 [CG2]	HC-FW1	NAG 154-2 [O7]	A	3.60	V 2 [CG1]	HC-FW1
	BMA 154-1 [O6]	B	3.25	V 2 [CG2]	HC-FW1	BMA 154-1 [O6]	B	3.44	V 2 [CG1]	HC-FW1					
	BMA 154-1 [C3]	B	3.62	S 56 [OG]	L2	MAN 154-1 [O4]	B	3.26	K 45 [NZ]	LC-FW2	MAN 154-1 [O4]	A	3.26	K 45 [CD]	LC-FW2

Supplementary Table 2. Binding interaction for DenV3 sE sc30 - F25.S02 Fab and ZikV sE - F25.S02 Fab. Interactions of F25.S01 Fab and the sE proteins of DenV3 and ZikV as computed by the PDBePISA server [1]. Hydrogen bonds involving Fab sidechains are highlighted in green and hydrogen bonds involving Fab mainchain are in red. Salt bridges are highlighted in yellow. Hydrogen bonds distances cutoff: 3.5 Å, salt bridge distance cut off: ≤4 Å.

References

1. Krissinel E, Henrick K. Inference of macromolecular assemblies from crystalline state. J Mol Biol. 2007;372(3):774-97. Epub 20070513. doi: 10.1016/j.jmb.2007.05.022. PubMed PMID: 17681537.
