# Supplementary material for: Structural basis for antibody cross-neutralization of Dengue and Zika viruses
Source: Commun Biol. 2026 Mar 10;9:568. doi: 10.1038/s42003-026-09805-6 (PMC13106624; doi:10.1038/s42003-026-09805-6)
Supplement: Supplementary file 3 — Description of Additional Supplementary files [file 42003_2026_9805_MOESM3_ESM.pdf]

Description of Additional Supplementary files

File name: Supplementary Data 1

Description: The raw source data for Fig 2f, Fig 3c, Fig S3 and uncropped gels for Fig S1b,f.
